# Severe and fatal neonatal infections linked to a new variant of echovirus 11, France, July 2022 to April 2023

**DOI:** 10.2807/1560-7917.ES.2023.28.22.2300253

**Published:** 2023-06-01

**Authors:** Mathilde Grapin, Audrey Mirand, Didier Pinquier, Aurélie Basset, Matthieu Bendavid, Maxime Bisseux, Marion Jeannoël, Bérengère Kireche, Manoelle Kossorotoff, Anne-Sophie L’Honneur, Lila Robin, Yves Ville, Sylvain Renolleau, Véronique Lemee, Pierre-Henri Jarreau, Isabelle Desguerre, Florence Lacaille, Marianne Leruez-Ville, Clémence Guillaume, Cécile Henquell, Alexandre Lapillonne, Isabelle Schuffenecker, Mélodie Aubart

**Affiliations:** 1 Paediatric Intensive Care Unit, Necker-Enfants malades University Hospital, Assistance Publique Hôpitaux de Paris, Paris Cité University, Paris, France; 2 Clermont-Ferrand University Hospital, 3IHP - Infection Inflammation et Interaction Hôtes Pathogènes Virology Department, French Reference Centre for enteroviruses and parechovirus, coordination laboratory, Clermont-Ferrand, France; 3 Auvergne University, LMGE UMR CNRS 6023, Team Epidemiology and pathophysiology of enterovirus Infection, Clermont-Ferrand, France; 4 Neonatal and Paediatric Intensive Care Units, Rouen University Hospital, Rouen, France; 5 Neonatal Intensive Care Unit, Cochin-Port Royal University Hospital, Assistance Publique Hôpitaux de Paris, Paris Cité University, Paris, France; 6 Hospices Civils de Lyon, Virology Department, French Reference Centre for enteroviruses and parechoviruses, associated laboratory, Lyon, France; 7 Neonatal and Paediatric Intensive Care Units, Orléans Regional Hospital, Orléans, France; 8 Paediatric Neurology Department, Necker-Enfants malades University Hospital, Assistance Publique Hôpitaux de Paris, Paris Cité University, Paris, France; 9 Virology laboratory, Cochin University Hospital, Assistance Publique Hôpitaux de Paris, Paris Cité University, Paris, France; 10 Obstetrics and Fetal Medicine Department, Necker-Enfants malades University Hospital, Assistance Publique Hôpitaux de Paris, Paris Cité University, Paris, France; 11 Virology Department, Rouen University Hospital, Rouen, France; 12 Pediatric Gastroenterology-Hepatology-Nutrition Unit, Necker-Enfants malades University Hospital, Assistance Publique Hôpitaux de Paris, University of Paris, Paris, France; 13 Clinical Microbiology laboratory and Virology unit, Necker-Enfants malades University Hospital, Assistance Publique Hôpitaux de Paris, Paris Cité University, Paris, France; 14 Orléans Hospital, Microbiology laboratory, Orléans, France; 15 Neonatal Intensive Care Unit, Necker-Enfants malades University Hospital, Assistance Publique Hôpitaux de Paris, Paris Cité University, Paris, France; 16 Laboratory of Human Genetics of Infectious Diseases, Necker Branch, Imagine Institute, Institut National de la Santé et de la Recherche Médicale U1163, Paris, France; * These authors contributed equally to the work and share the first authorship.; ** These authors contributed equally to the work and share the last authorship.

**Keywords:** Enterovirus, Echovirus-11, neonatal sepsis, liver failure

## Abstract

We report nine severe neonatal infections caused by a new variant of echovirus 11. All were male, eight were twins. At illness onset, they were 3–5 days-old and had severe sepsis and liver failure. This new variant, detected in France since April 2022, is still circulating and has caused more fatal neonatal enterovirus infections in 2022 and 2023 (8/496; 1.6%, seven associated with echovirus 11) compared with 2016 to 2021 (7/1,774; 0.4%). National and international alerts are warranted.

Enteroviruses (EV) are a common cause of neonatal infections with clinical manifestations ranging from asymptomatic to severe and sometimes fatal disease [1–3]. Between July 2022 and April 2023, nine cases of severe neonatal infection with a liver failure were reported in France. Seven of these children died. All were associated with a new variant of echovirus 11 (E-11). We describe the clinical and virological characteristics of this upsurge of highly severe neonatal EV infections.

## Description of the cases

Between July 2022 and April 2023, nine children from three French metropolitan regions were hospitalised in paediatric intensive care units for suspected neonatal sepsis. A description of each case is available in Supplement 1. All patients were male. Only one was born at full term and was from a singleton pregnancy. The other eight patients were from twin pregnancies. They were born between 31 weeks and 5 days and 36 weeks and 3 days of gestation and were initially hospitalised in neonatology departments before developing the first symptoms (Table 1).

**Table 1:** Clinical characteristics of enterovirus-associated severe neonatal infections of male neonates with acute liver failure and multivisceral failure, France, 2022–2023 (n  =  9)

Patient number	Gestation length (weeks + days)	Date of birth	Mode of delivery	Birth weight (g)	Age at first symptoms (days)	Specimen type	Age at diagnosis of EV infection (days)	Maternal EV infection	Maternal EV infection (days from delivery)	Treatment	Dialysis	Age at death (days)
1^a^	39 + 0	Jan 2023	Vaginal	3,480	5	CSF	5	Yes	0	Pocapavir D7-D21 IVIg D5	Yes	34
2	35 + 4	Dec 2022	Caesarian	2,400	5	Blood	5	Yes	-3^b^	IVIg D7, D8	Yes	19
3				2,650		Blood				IVIg D7, D8	Yes	40
4	31 + 5	Oct 2022	Vaginal	1,805	5	Blood	6	NA	NA	No	No	7
5				2,320		Post-mortem biopsies				No	No	6
6	36 + 3	Jul 2022	Vaginal	2,600	3	CSF	3	Yes	-3^b^	IVIg D4	No	5
7				2,860		CSF				IVIg D3, D4	No	5
8	34 + 0	Mar 2023	Vaginal	2,375	4	CSF	8	Yes	-1^b^	Pocapavir D8-D23 IVIg D18, 19	No	NA
9				1,970		CSF				Pocapavir D8-D23 IVIg D18, 19	No	NA

CSF: cerebrospinal fluid; IVIg: polyvalent intravenous immunoglobulins; NA: not applicable.

^a^ From a singleton pregnancy.

^b^ Days before delivery.

In all patients, the first symptoms appeared between the third and fifth day of life. The initial symptoms were fever and apnoea, which were rapidly followed by signs of septic shock requiring active resuscitation including administration of amines, with the exception of Patients 8 and 9, who responded to vascular filling alone. All cases developed acute hepatocellular failure with severe cytolysis and disseminated intravascular coagulation as early as the first day of hospitalisation. Blood coagulation factor V, II, VII, X, Quick factor, and fibrinogen were undetectable in all patients. The patients had hyperammonaemia: five were treated with sodium benzoate and three with haemodialysis and one died before treatment. All patients had thrombocytopaenia, and seven patients required transfusions of platelets, plasma, and tranexamic acid to control the haemorrhagic syndrome. All cases had acute renal failure at the onset of the symptoms, one had myocarditis, three were diagnosed with meningoencephalitis and two had enterocolitis. Transfontanellar ultrasound was performed for all patients: five of them had normal findings, two had multiple hyperechogenic lesions in the white matter, confirmed by cerebral magnetic resonance imaging (MRI), and two had bilateral intraventricular haemorrhage, grade III.

## Bacteriological and virological investigations

Bacterial blood cultures were negative in seven patients within 48 h of the onset of the symptoms. Blood culture was positive for *Escherichia coli* in one neonate and for *Staphylococcus epidermidis* in another. The EV genome was detected in all available specimens, including blood, cerebrospinal fluid, throat, nasopharyngeal or rectal swabs, stool, post-mortem biopsies or dried blood spots (Table 2). We identified E-11 as the causative agent in all cases either by next generation sequencing of (sub)-complete genomes using direct RNA sequencing or amplicon sequencing adapted from the previously described method in Duval et al. [4]), or by Sanger sequencing of the 1D gene coding the VP1 capsid protein [5,6].

**Table 2:** Virological data on the echovirus-11-infected severe cases and their mothers, France, 2022–2023

Patient	Sample type	Age of the child at sampling (days)	Sequence	Accession number
1	Dried blood spot	3	Not typeable	NA
	CSF	5	Complete 1D^VP1^	OQ927993
	Stool	6	Complete genome	OQ927998
	Plasma	6	Complete genome	OQ923264
Mother 1	Serum	0	Complete 1D^VP1^	OQ927994
	Milk	9	Partial 1D^VP1^	Not deposited
2	Dried blood spot	3	Partial 1D^VP1^	Not deposited
	Plasma	5	Complete genome	OQ927999
3	Dried blood spot	3	Partial 1D^VP1^	Not deposited
	Plasma	5	Complete genome	OQ928000
Mother 2–3	Serum	D-3^a^	Complete 1D^VP1^	OQ927995
4	Dried blood spot	3	Partial 1D^VP1^	Not deposited
	Psoas muscle biopsy	6	Complete 1D^VP1^	OQ927996
	Liver biopsy	6	Complete genome	OQ928001
	Lung biopsy	6	Complete genome	OQ928002
5	Dried blood spot	2	Partial 1D^VP1^	Not deposited
	Blood	6	Complete genome	OQ927567
6	CSF	3	Complete genome	OQ928003
7	CSF	3	Complete genome	OQ928004
Mother 6–7	Serum	1	Complete 1D^VP1^	OQ927997
8	Dried blood spot	3	Partial 1D^VP1^	Not deposited
	NP swab	8	Partial 1D^VP1^	Not deposited
	Throat swab	8	Complete genome	OQ969164
	Rectal swab	8	Complete genome	Not deposited (identical to OQ969164)
	Plasma	10	Complete 1D^VP1^	Not deposited
9	Dried blood spot	3	Partial 1D^VP1^	Not deposited
	NP swab	8	Partial 1D^VP1^	Not deposited
	Throat swab	8	Complete genome	OQ969165
	Rectal swab	8	Complete genome	Not deposited (identical to OQ969165)
	Plasma	10	Complete 1D^VP1^	Not deposited
Mother 8–9	Serum	D-1^a^	Complete 1D^VP1^	OQ971926

CSF: cerebrospinal fluid; D: delivery; ND: not determined; NP: nasopharyngeal; VP1: capsid protein; 1D: gene coding for VP1 capsid protein.

^a^ Samples collected before delivery.

Maternal blood samples collected during the peripartum period were available for four of the five mothers with gastrointestinal symptoms or fever during the 3 days before or at delivery. All were EV-positive with subsequent identification of an E-11.

All patients received empiric antibiotic therapy at the onset of clinical symptoms. Treatment for EV infection included polyvalent intravenous immunoglobulins (IVIg) in seven patients and pocapavir for 14 days in three patients (Table 1). Seven of the nine patients died: four without a dialysis between 5 and 7 days of life and three between 19 and 40 days of life. A pair of twins (Patients 8 and 9) survived with no evidence of sequelae at the age corrected to term birth.

## Molecular characterisation of a new E-11 variant

**Figure 1:**
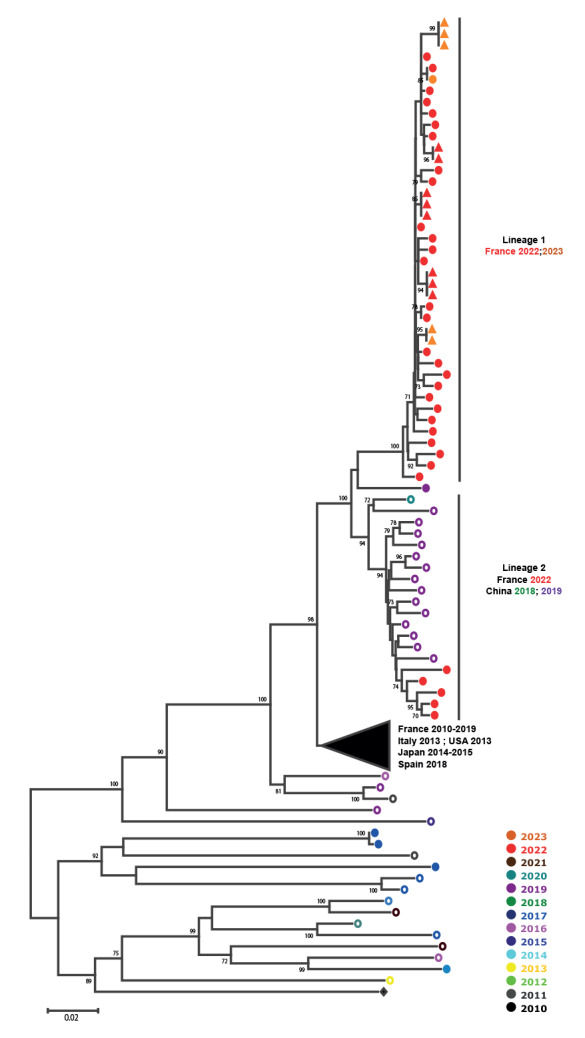
Phylogenetic tree of echovirus 11 complete 1D^VP1^ sequences from neonatal and non-neonatal infections in France and other countries, 2010–2023 (n = 142)

**Figure 2:**
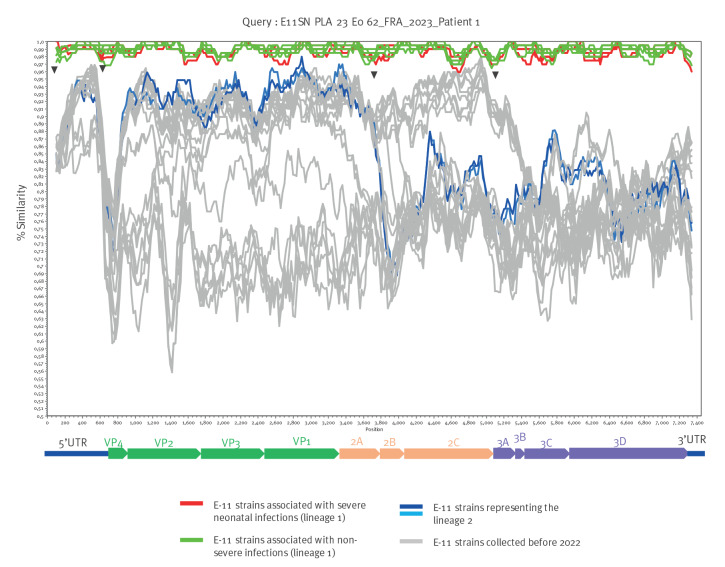
Analysis of nt similarity of echovirus 11 complete genome sequences from neonatal and non-neonatal infections, France and other countries, 2010–2023 (n = 38)

Phylogenetic analyses were performed on complete 1D^VP1^ E-11 sequences determined from specimens collected in France between 2010 and 2023 from patients (n  =  104) in the five hospitals from where the severe neonatal cases were reported, as well as sequences from other countries for which complete genome sequences were available from GenBank (n  =  37). E-11 strains detected in 2022 and 2023 fell into two separate lineages, distinct from the E-11 strains isolated before 2022 (Figure 1). The lineage 1 included all E-11 sequences associated with severe neonatal infections as well as sequences from non-severe neonatal and non-neonatal infections. The remaining French sequences from 2022 were grouped in lineage 2 and were related to strains isolated in China in 2018 and 2019. Similarity plot analysis of the E-11 complete genome sequences suggested a recombinant origin for both lineages. The lineage 1 representing genomes appears to be a mosaic defined by distinct similarity patterns characterised by sharp decrease in nt similarity within the 5’-untranslated region and in the 3A coding region, possibly arising through recombination (Figure 2) compared with E-11 strains collected before 2022. They also differed from lineage 2 complete genomes whose similarity plots are characterised by a recombination signal in the 2A coding region.

MEGA: molecular evolutionary genetics analysis.

The phylogenetic tree was constructed by the neighbour joining method and evaluated with 1,000 bootstrap pseudoreplicates, using MEGA6. Only bootstrap values > 70% are indicated. Genetic distances were calculated with Tamura-Nei’s model of evolution and branch length is drawn to the indicated scale (proportion of nt substitution per site). The strains collected in patients with severe neonatal infection are labelled with a filled triangle. Strains collected in France (n = 104) between 2010 and 2023, accession numbers OQ927567, OQ923264, OQ927993–927997, OQ927998–928004, OQ969158-OQ969177, OQ971926-OQ971949; OR029978–030028) and in other countries (n = 37) (selected sequences among complete E-11 genomes available in GenBank, as of 28 April 2023) are labelled with a filled circle or empty circle, respectively. Year of isolation is colour coded. For clarity, the taxon names are not indicated in the tree. See Supplement 2 for taxon names. The French sequences from 2010 to 2023 were detected from samples from hospitalised patients in Orléans, Lyon, Clermont-Ferrand, Rouen, Cochin-Port Royal (Paris) and Necker-Enfants Malades (Paris) Hospitals, where the severe cases were reported.

UTR: Untranslated region.

Complete genome sequences of the French E-11 strains of 2022–2023 (n = 26) were compared with each other and with other E-11 strains (11 from other countries and the prototype strain X.80059) using SimPlot 3.5.1. A schematic diagram of the enterovirus genome is shown at the bottom of the panel. Recent E-11 genomes, including those of the strains associated with severe neonatal infection or non-severe infection within the lineage 1 and the other French strains from 2022 to 2023 (lineage 2) are represented in red, green and blue, respectively. The genomes of strains collected before 2022 are represented in grey. Potential recombination breakpoints are indicated by black arrows.

## National surveillance data

**Figure 3:**
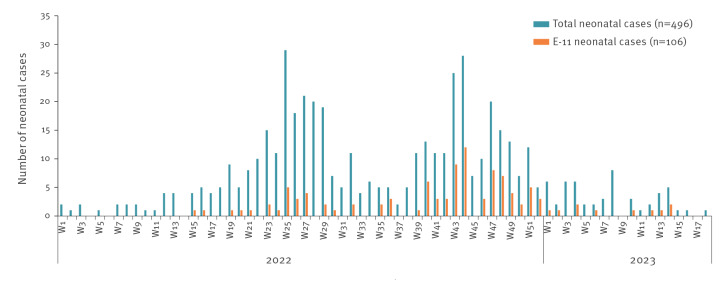
Distribution of neonatal cases by week, France, January 2022–April 2023 (n = 496)

The hospital-based EV surveillance in France is a voluntary system involving 36 virology or microbiology laboratories, covering approximatively 100 hospitals [7] distributed in all French regions, except Corsica, coordinated by the National Public Health Agency, Santé publique France, and the two National Reference Laboratories (NRLs). Participants report to the NRLs the number and type of samples analysed for EV and the relevant epidemiological and standardised clinical data of the EV-positive cases [8]. EV-positive samples are sent to the NRL laboratories for genotyping, whenever possible. Between January 2022 and April 2023, 2,026 EV cases were reported, among which neonatal cases represented 24.5% of the cases. Echovirus-11 was the most predominant EV type identified among neonates (31.1%, 106 of 341) and non-neonates (15.3%, 158 of 1,035). The epidemic curve (Figure 3) shows that E-11 has been continuously detected since April 2022. 

In 2022–23, 28 (5.6%) of the 496 neonatal cases presented with severe EV infection (defined as at least one organ failure requiring intensive care support). We identified E-11 in 14 of the 26 cases with a known type, among which nine had severe sepsis with liver failure and five had apnoea requiring high-flow oxygen therapy. By comparison, from 2016 to 2021, 62 (3.5%) severe neonatal infections were reported, and E-11 was identified in 6.2% of the cases with a known type (3/48). Moreover, a higher mortality was observed in 2022 and 2023 among the EV neonatal cases: eight (1.6%) of the 496 children died, all but one associated with E-11 compared with seven (0.4%) of 1,774 in 2016–2021, none associated with E-11.

Clinical presentation of other E-11 infections in children did not seem to have changed during this period. Finally, male children were overrepresented among the severe E-11 neonatal cases (12/14) and among the severe EV neonatal cases (20/28), whereas the proportion of males among all EV neonatal cases was 56.3% (276/490).

## Discussion

We report a cluster of severe neonatal cases with liver failure and a high mortality rate associated with a new variant of E-11, leading to an alert in the European Union Early Warning and Response System on 4 May 2023, and inclusion in the ECDC Communicable Disease Threat Report [9]. Enterovirus infections in neonates may be associated with severe clinical illness and mortality depending on (i) the infecting EV type, of which the group B coxsackieviruses and E-11 are the most commonly EVs associated with neonatal sepsis [10] and (ii) the clinical infection phenotype, where the mortality rate is the highest for severe hepatitis or myocarditis manifestations [11].

Fulminant hepatitis associated with E-11 has already been described, but the high fatality rate within a nine-month period and the high proportion of twin cases were striking in this cluster. All patients presented with one or more well-known risk factors for severe neonatal EV infection, which are maternal evidence of a recent EV infection (four of the five mothers in this report), prematurity and onset of illness within the first few days of life [1,11].

The emergence of a new E-11 variant of recombinant origin in 2022, after a period of a low-level circulation of this type since 2008 may have led to a higher proportion of women susceptible to E-11 infection and subsequently to the higher proportion of E-11 associated neonatal infections observed in 2022–2023. Recombination, which frequently occurs in enteroviruses, is considered a factor driving viral emergence. Whether the genetic changes of the predominant circulating E-11 lineage have influenced the pathogenicity needs further investigation. Host genetic factors affecting immunity might also have influenced the clinical severity observed in the nine cases (by comparison with other patients infected with the same E-11 variant during their first week of life) as previously described in EV rhombencephalitis cases [12]. High prevalence of boys in severe neonatal EV infection might speak for a predisposition associated with X-chromosome.

Therapeutic options for neonatal EV infections are limited and include intravenous immunoglobulins or investigational/compassionated specific antiviral therapy consisting of pleconaril or pocapavir [13–15]. We used pocapavir for three patients, of whom two survived, but the efficacy of the treatment cannot be concluded from our data.

## Conclusion

This report suggests that a new variant of E-11 is currently circulating and associated with a high risk of severe neonatal infection and death at least in France. Clinicians should be aware of potential involvement of EV in severe clinical presentations in neonates, as they are at the frontline to detect such cases. Enterovirus surveillance in France, as in most European country, is a voluntary system reporting EV-positive cases which could lead to an underestimation of the number of severe cases if EV genome detection is not considered. As a reminder, symptomatic neonatal EV disease initially presents as a neonatal sepsis, which is clinically indistinguishable from bacterial or herpes simplex virus infections. Neonates with an unexplained sepsis who present with signs of myocarditis or liver failure with cytolysis should be rapidly evaluated for EV infection, especially if the mother has had acute symptoms of gastroenteritis in the days before birth. Blood, as well as respiratory, cerebrospinal fluid and stool samples should be collected for initial testing and further sequencing. Therapeutic options as IVIg or pocapavir should be considered.

## Supplementary Data

162000 Supplementary Material 1

## Supplementary Data

83000 Supplementary Material 2
