# Supplementary material for: Severe and fatal neonatal infections linked to a new variant of echovirus 11, France, July 2022 to April 2023
Source: Euro Surveill. 2023 Jun 1;28(22):2300253. doi: 10.2807/1560-7917.ES.2023.28.22.2300253 (PMC10236930; doi:10.2807/1560-7917.ES.2023.28.22.2300253)
Supplement: Supplementary Material 1 [file 23-00253_AUBART_Supplement_1.pdf]

This supplementary material is hosted by *Eurosurveillance* as supporting information alongside the article' Severe and fatal neonatal infections linked to a new variant of echovirus 11, France, July 2022 to April 2023', on behalf of the authors, who remain responsible for the accuracy and appropriateness of the content. The same standards for ethics, copyright, attributions and permissions as for the article apply. Supplements are not edited by *Eurosurveillance* and the journal is not responsible for the maintenance of any links or email addresses provided therein.

Case report:

Patient 1:
Patient 1 was born at 39 weeks of amenorrhea with a weight of 3480g, at birth the APGAR score was 10/10. His mother had fever during delivery without any other symptom. He left maternity hospital normally at 3 days of life. At 5 days, he came to emergency for a fever at 38.7°C. An inflammatory syndrome was found with a CRP at 47 mg/L, procalcitonin at 9.5μg/L. Cytochemical parameters of the cerebrospinal fluid were normal. Enterovirus (EV) RT-PCR was positive in the CSF but also in plasma and stools. Maternal blood collected at birth was retrospectively found positive for EV genome detection as well as milk collected at 9 days post-birth. EV genotyping identified an E-11.
He was first treated with cefotaxime, gentamycin and aciclovir in pediatric unit. He quickly presented an hemorrhagic syndrome at each puncture site and hypoglycemia. He had thrombopenia at 11000/mm3 and hepatic cytolysis. He was then transferred to pediatric intensive care unit where he was intubated at arrival for apnea. He developed acute hepatocellular failure with a major cytolysis (Aspartate aminotransferase [AST] 8500 IU/L, Alanine aminotransferase [ALT] 680 IU/L) and disseminated intravascular coagulation. Factors V, II, VII, X, Quick factor and fibrinogen were undetectable. He received daily transfusions of platelets, plasma, tranexamic acid to control hemorrhage. He presented acute renal failure on hepato-renal syndrome with an important overload and hyperammoniemia requiring dialysis and noradrenalin.
EV infection was treated with polyvalent immunoglobulins at day 5 and pocapavir from day 7 to day 21 (14 days of treatment). The EV viral load decreased from very high to medium in 8 days.
He was extubated at day 15 and reintubated at day 17 for intra-alveolar hemorrhage. He had a slight improvement of hepatic function at day 23 with a factor V at 20% and a normalization of the cytolysis but still an increase of total bilirubin max at 715 μmol/L and a conjugate at 350 μmol/L. Cerebral MRI was performed at day 24 and found multiple T1 hypersignal lesions in bilateral supratentorial location, subcortical and deep periventricular white matter distribution. He had a second attempt of extubation at day 25 but was reintubated for multivisceral failure and intra-alveolar hemorrhage at day33. He died at day 34.

Patient 2 and patient 3:
They were born at 35+4 weeks of amenorrhea from a biamniotic monozygotic twin pregnancy through a cesarean section of scar uterus. Patient 2 weighed 2650g and patient 3 2400g. Both children were hospitalized in a neonate unit.
Their mother and an older child had gastroenteritis symptoms the day before birth.
Initially they both had the same clinical presentation. They presented at day 5 a shock without response to vascular filling requiring hydrocortisone and noradrenalin. They were both

intubated at day 5. They developed acute hepatocellular failure with a major cytolysis (patient 2: AST 9000 IU/L, ALT 1217 IU/L, patient 3: AST 13000 IU/L; ALT 1700 IU/L) and disseminated intravascular coagulation. Factors V, II, VII, X, quick factor and fibrinogen were undetectable and they had thrombopenia (platelets 11000/mm3 and 10000/mm3) requiring daily transfusions of platelets, plasma and continuous perfusion of tranexamic acid. They were treated by cefotaxime and gentamycin before virological results.
EV RT-PCR was found positive in blood, stool, nasopharygeal sample. Maternal blood collected 3 days before birth was retrospectively found positive for EV genome detection. EV genotyping identified an E-11.
EV infection was treated with polyvalent immunoglobulins at D7 and D8. Sodium benzoate administration could not control the increase of hyperammonemia.
They both developed acute renal failure on hepato-renal syndrome. Dialysis started on D11 for overload fluid and hyperammonemia.
EV viral load decreased spontaneously from very high at D5 to very low at D18.
Patient 3 presented at D17 acute multivisceral failure with a choc requiring noradrenalin, adrenalin, vasopressin, an ARDS with refractory hypoxemia without response to NO 20ppm, uncontrolled hemorrhage and cardiac dysfunction. He died at D19.
Patient 2 presented a spontaneous decrease of cytolysis with an increase of total bilirubin up to 623 μmol/L. A cerebral MRI was realized at D29, which found multiple bleeds in the periventricular white matter without SWAN hyposignal, the largest being 9mm in the left frontal area. No clear cortical ribbon abnormality. T2 and T1 hypersignals in the basal ganglia, without visible bleeding. In view of the severity of the situation, and in particular the neurological picture, the decision was made to withdraw active therapies, extubation at D33, death at D40.

Patient 4 and patient 5:
They were born at 31+5 weeks of amenorrhea from a biamniotic monochorionic twin pregnancy. Patient 4 weighed 2320g and patient 3 1805g. Both children were hospitalized in neonate unit. They both presented refractory shock requiring dobutamine max at 20μg/kg/min and noradrenaline at 2 μg/kg/min.
Patient 4 presented an ulceronecrosing enterocolitis with pneumatosis. He was treated with vancomycin, gentamycin, cefotaxime and metronidazole, then meropenem at D4. He then developed a multivisceral failure with acute renal failure and thrombopenia at 10 000/mm3. No liver biological testing was obtained. He died at D6 of a cardiac arrest.
Enterovirus RT-PCR was not performed in patient 4 but it was positive in patient 5 with a high viral load. Post-mortem biopsies were retrospectively screened for EV genome in patient 4 and were positive. EV genotyping identified an E-11 for both patients.
Patient 5 was also treated with vancomycin, gentamycin andcefotaxime, then meropenem. He presented suddenly a new deterioration at D6 with acute hepatocellular failure, major cytolysis (AST 489500 IU/L, ALT 62870 IU/L) and disseminated intravascular coagulation. Factors V, II, VII, X, Quick factor were undetectable and he had thrombopenia (platelet counts of 14×10^9/L). He died at D7.

Patient 6 and patient 7:
They were born at 36+3 weeks of amenorrhea from a biamniotic bichorionic twin pregnancy. Patient 6 weighed 2860g and patient 7 2600g. Their mother had gastroenteritis symptoms 3 days before borth. Both boys were hospitalized in neonate unit. They both presented

hypotonia and cyanosis at D3 and rapidly a shock with hyperlactatemia and disseminated intravascular coagulation. Factors V, II, VII, X, quick factor and fibrinogen were undetectable and they had thrombopenia. They required transfusion of platelets and plasma. They were treated with amoxicillin, gentamycin and polyvalent immunoglobulins from D3 to D4. EV RT-PCR was positive in CSF for both patients; an E-11 was identified. They both died at D5 from multivisceral failure. Maternal blood collected at birth retrospectively was positive for EV.

Patients 8 and patient 9:

They were boys, born at 34 weeks of amenorrhea from a biamniotic monochorionic twin pregnancy. Patient 8 weighed 1970g and patient 9 2375 g. Both children were hospitalized in neonate unit. They both presented at D4 apneaand hypotonia treated with cefotaxime, vancomycin, amikacin and non-invasive ventilation with CPAP. Patient 8 presented an ulceronecrosing enterocolitis confirmed using abdominal X-rays. They both had EV meningitis with positive RT-PCR in CSF. They rapidly developed acute hepatic failure with cytolysis (patient 8: AST 2442 IU/L, ALT 477 IU/L; patient 9: AST 3650 IU/L, ALT 988 IU/L) disseminated intravascular coagulation at D8 and required transfusion of platelets and plasma. Coagulation factors were low with Quick factor minimal below 10%, V factor at 13% for patient 8 and Quick factor below 10% and V factor at 9% for patient 9. Fibrinogen was not obtained for both patients. They developed acute renal failure with overload fluid that respond to furosemide and spironolactone. They had hyperammonemia controlled by benzoate sodium.

they were treated with pocapavir between D8 and D23 and they received polyvalent immunoglobulins at D18 and D19. EV RT-PCR which was still positive at D25 for both twins. Patient 9 had an adrenal hematoma on the last abdominal ultrasound. Transfontanelar ultrasound were normal during the hospitalization. At D30 children were still alive, Quick factor was 30% for patient 8 and 42% for patient 9,.They still had cytolysis with AST 110 IU/L, ALT 21 IU/L for patient 8 and AST 97 IU/L, ALT 17 IU/L, total bilirubin level was 198 μmol/L for patient 8 and 155 μmol/L for patient 9. The 2 children survived with no evidence for sequelae at birth term age.
