# Supplementary material for: Severe and fatal neonatal infections linked to a new variant of echovirus 11, France, July 2022 to April 2023
Source: Euro Surveill. 2023 Jun 1;28(22):2300253. doi: 10.2807/1560-7917.ES.2023.28.22.2300253 (PMC10236930; doi:10.2807/1560-7917.ES.2023.28.22.2300253)
Supplement: Supplementary Material 2 [file 23-00253_AUBART_Supplement_2.pdf]

This supplementary material is hosted by *Eurosurveillance* as supporting information alongside the article' Severe and fatal neonatal infections linked to a new variant of echovirus 11, France, July 2022 to April 2023', on behalf of the authors, who remain responsible for the accuracy and appropriateness of the content. The same standards for ethics, copyright, attributions and permissions as for the article apply. Supplements are not edited by *Eurosurveillance* and the journal is not responsible for the maintenance of any links or email addresses provided therein.

Suppl Figure 1

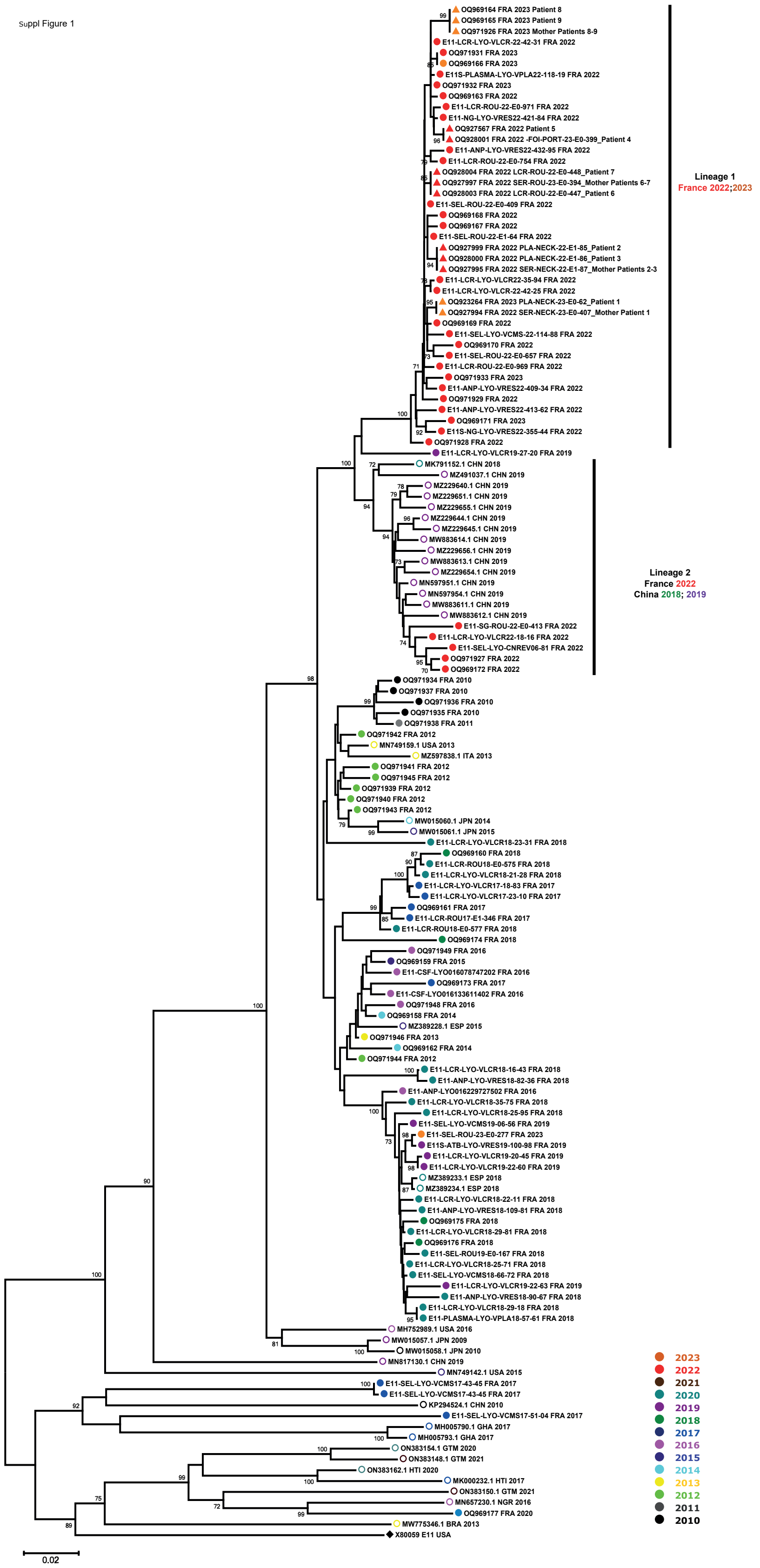

The phylogenetic tree was constructed by the neighbour joining method and evaluated with 1,000 bootstrap pseudoreplicates, using MEGA6. Only bootstrap values >70% are indicated. Genetic distances were calculated with Tamura-Nei's model of evolution and branch length is drawn to the indicated scale (proportion of nucleotide substitution per site). The strains collected from patients with severe neonatal infections are labelled with a filled triangle. Strains collected in France (n = 104) between 2010 and 2023, accession numbers OQ927567, OQ923264, OQ927993–927997, OQ927998-928004, OQ969158-OQ969177, OQ971926-OQ971949; OR029978-030028) and in other countries (n = 37) (selected sequences among complete E-11 genomes available in GenBank, as of 28 April 2023) are labelled with a filled circle or empty circle, respectively. Year of isolation is colour coded. The French sequences from 2010 to 2023 were from samples from hospitalised patients in Orléans, Lyon, Clermont-Ferrand, Rouen, Cochin-Port Royal (Paris) and Necker-Enfants Malades (Paris) Hospitals, where the severe cases were reported.
